# An evaluation of phase angle, bioelectrical impedance vector analysis and impedance ratio for the assessment of disease status in children with nephrotic syndrome

**DOI:** 10.1186/s12882-019-1511-y

**Published:** 2019-08-22

**Authors:** Steven Brantlov, Lars Jødal, René Frydensbjerg Andersen, Aksel Lange, Søren Rittig, Leigh C. Ward

**Affiliations:** 1 Department of Procurement and Clinical Engineering, Aarhus University Hospital, Central Denmark Region, Olof Palmes Allé 15, 8200 Aarhus, Denmark; 2 Department of Nuclear Medicine, Aalborg University Hospital, Aalborg, Denmark; 3 Department of Paediatrics and Adolescent Medicine, Aarhus University Hospital, Aarhus, Denmark; 4 School of Chemistry and Molecular Biosciences, The University of Queensland, Brisbane, Australia

**Keywords:** Electrical impedance, Phase angle, Bioelectrical impedance vector analysis, Impedance ratio, Total body water, Nephrotic syndrome, Children

## Abstract

**Background:**

Oedema, characterized by accumulation of extracellular water (ECW), is one of the major clinical manifestations in children suffering from nephrotic syndrome (NS).

The lack of a simple, inexpensive and harmless method for assessing ECW may be solved by the use of the bioelectrical impedance analysis (BIA) technique.

The aims of this study were to examine whether phase angle (PA), bioelectrical impedance vector analysis (BIVA) and the impedance ratio (IR) reflect change in disease status in children with NS.

**Methods:**

Eight children (age range: 2–10 years) with active NS (ANS group) were enrolled. In five of these (ANS* subgroup), impedance was also measured at remission (NSR group). Thirty-eight healthy children (age range: 2–10 years) were included as healthy controls (HC group). Whole-body impedance was measured with a bioimpedance spectroscopy device (Xitron 4200) with surface electrodes placed on the wrist and ankle.

**Results:**

Values of PA, BIVA and IR were found to be significantly lower (*p*-value range < 0.001 to < 0.01) in the ANS patients compared to the HC and NSR groups. No significant differences were observed between the NSR and HC groups.

**Conclusion:**

The studied parameters can be used to assess change in disease status in NS patients. Data were consistent with NS being associated with expansion of ECW.

**Electronic supplementary material:**

The online version of this article (10.1186/s12882-019-1511-y) contains supplementary material, which is available to authorized users.

## Background

Nephrotic syndrome (NS) is a condition in which the kidneys leak large amounts of proteins into the urine, with consequent hypoalbuminemia and oedema formation [1] and thus causes increased risk of complications and prolonged hospitalization [2].

Normally, body weight measurement is used by clinicians as a measure of oedema, i.e., accumulation of extracellular water (ECW), in NS patients. However, the challenge with this approach is that it is only reliable for short periods of time during which the change in weight due to causes other than water are non-significant [3].

Dilution techniques using the tracers deuterated water (D_2_O, “heavy water”) and sodium bromide (NaBr) are the criterion techniques for determining total body water (TBW) and ECW volumes respectively. These are, however, potentially invasive, expensive, time consuming, not possible to repeat at short intervals and require highly trained personnel to make the measurements [4], making them unsuitable for routine use in the clinic [5].

For these reasons, there is need for a simple, inexpensive and harmless method for routine assessment of disease status that can provide new and clinically useful information to clinicians in the treatment of pediatric NS patients. A possible approach that may prove to be of use is the bioelectrical impedance analysis (BIA) technique, which is characterized by being non-invasive, harmless, quick, simple, inexpensive, and portable and thus suitable for routine use [6].

BIA, which is a collective term for bioimpedance devices used to determine body composition [7], is based on the principle that the flow of alternating electrical current through the body varies from tissue to tissue; tissues containing large amounts of water and electrolytes have high conductivity, i.e., low impedance, whereas fat and bone have low conductivity and correspondingly high impedance. The impedance of the body is therefore quantitatively (inversely) related to the volume of water in the body.

However, a challenge with BIA in paediatric populations is that calculation of body water volumes are based on population specific [8, 9] prediction equations that may be inaccurate [10, 11]. Therefore, there is a growing interest in the use of raw impedance data, especially in patients with altered body water distribution [12]. Raw impedance data are not directly linked to body water volumes, but can be considered as indices of such volumes that can vary with sodium and water retention.

Of the BIA parameters using raw impedance data, phase angle (PA) is the most commonly used [13]. PA has been linked with body water distribution between the intracellular water (ICW) and ECW spaces (tissue hydration) and cell membrane integrity (amount and quality) in a number of studies [14–16], but the exact biological meaning of the parameter is still not fully understood [13].

Another approach is bioelectrical impedance vector analysis (BIVA), which is based on pattern analysis of the raw impedance data standardized by the subject’s height, plotted as a bivariate vector in a nomogram [17]. This one has gained increased attention in the clinical setting [13], for example with studies conducted in healthy [18] and diseased [19, 20] pediatric populations.

A third approach is the impedance ratio (IR), which has been proposed as a potential indicator of oedema or overall health [12] and as a predictor of mortality in hemodialysis patients [21]. Moreover, IR has been suggested to reflect the fluid distribution between the TBW and the ECW [12]. Until now, few studies using raw impedance data have been performed in adults and none in pediatric populations [12, 22].

The main aim of the present study was to investigate whether PA, BIVA, and/or IR reflect change in disease status in pediatric patients with NS either active or in remission, and compare to results from healthy children. The BIA parameters are used as indices, while no attempt is made to quantify the fluid volumes. A secondary aim was to determine if there was advantage in using BIA parameters determined at the characteristic frequency, which is considered the optimal frequency for TBW measurement, rather than the conventionally-used 50 kHz.

## Methods

The study groups and parameters considered are summarized in Table 1 and described in the following.

**Table 1:** Groups and impedance parameters studied in this paper

Abbreviation	Description
ANS	Children with active NS (*n* = 8)
NSR	Children from ANS group re-studied at time of NS remission (*n* = 5)
ANS*	Subgroup of ANS, same children as NSR group (*n* = 5)
HC	Healthy controls, age-matched to ANS group (*n* = 38)
H	Patient height (m)
*f*_c_	The characteristic frequency (kHz)
R	Resistance (Ohm, Ω)
X_C_	Capacitive reactance (Ω)
PA_50_	Phase angle (in degrees) calculated at a frequency of 50 kHz
PA_*f*c_	PA calculated at *f*_c_
BIVA_RXc_	Bioelectrical impedance vector analysis plot based on R/H and X_C_/H (unit Ω/m)
BIVA_Zs_	BIVA plots based on Z scores (no unit)
IR_200/5_	Impedance ratio (no unit) calculated at the frequencies 200 and 5 kHz
IR_*f*c/5_	IR calculated at the frequencies *f*_c_ and 5 kHz.

### Subjects enrolled

Eight children (boys = 7, girl = 1, 2–10 years of age) with active NS (ANS patient group) were included from the Department of Pediatrics and Adolescent Medicine, Aarhus University Hospital, Denmark. Inclusion criteria were the presence of NS defined by proteinuria > 40 mg/m^2^/day, plasma albumin < 25 g/L, oedema, and hyperlipidemia. Exclusion criteria were low plasma levels of C3-complement, post-infectious glomerulonephritis, vasculitis such as Henoch-Schönlein nephritis or specific glomerulonephritis, e.g., dense deposit disease. Five of the ANS patients (ANS* subgroup) were also restudied on remission (NSR group). Remission was defined as urinary dipstick negative for protein on three consecutive days. Before treatment with prednisolone and diuretics were initiated, blood samples, blood pressures and impedance measurements were collected in the ANS patient group.

To be able to compare collected data, impedance measurements were also available for 38 healthy age-matched control children (HC group) (boys = 23, girls = 15, 2–10 years of age), taken from a previously published dataset [23].

### Anthropometry, biochemistry and blood pressure

Prior to the impedance measurements, weight and height were measured, in duplicate, by trained personnel. Weight was measured on digital scales, with light clothes to within 0.1 kg, and height was measured without shoes, to the nearest 0.5 cm using a stadiometer.

For all calculations, mean values were applied.

Resting venous blood samples and blood pressures (Carescape V100 Monitor, GE Healthcare, USA) were collected in the ANS patient group as part of their routine medical care with analyses undertaken by accredited hospital biomedical scientists.

### BIA parameters

#### Frequencies

Accepted practice is to use PA calculated at a frequency of 50 kHz [12, 13]. Similarly, BIVA is performed using resistance (R, in Ohm, Ω) and reactance (X_C_, in Ω) also measured at 50 kHz [13], while the IR in most common use is calculated from measurements of impedance at 5 and 200 kHz [12]. The use of a bioimpedance spectroscopy device, as used in this study, allows determination of the so-called characteristic frequency (*f*_c_, kHz), defined as the frequency at which X_C_ reaches its maximum [7]. This frequency (*f*_c_) varies from person to person, but can be considered the optimal frequency for measurement of TBW [24]. Consequently, it was decided to explore whether there was advantage in using measurements at *f*_c,_ in PA, BIVA and IR analyses, as an alternative to the accepted practice described above.

#### Phase angle (PA)

PA (in degrees), was calculated from R and X_C_ (Additional file 1: Figure S1) at both 50 kHz (PA_50_) and *f*_c_ (PA_*f*c_) by use of the following formula:

1 $$ \mathrm{PA}={\tan}^{-1}\left(\frac{{\mathrm{X}}_{\mathrm{C}}}{\mathrm{R}}\right)\bullet \frac{180}{\uppi} $$

In this formula, tan^−1^ transforms the ratio X_C_/R into an angle measured in radians, while the factor 180°/π (≈ 57.296) converts radians to degrees (2π in radians is a full circle, i.e., 360°). A larger ratio of X_C_/R corresponds to a larger PA.

#### BIVA

**Fig. 1:**
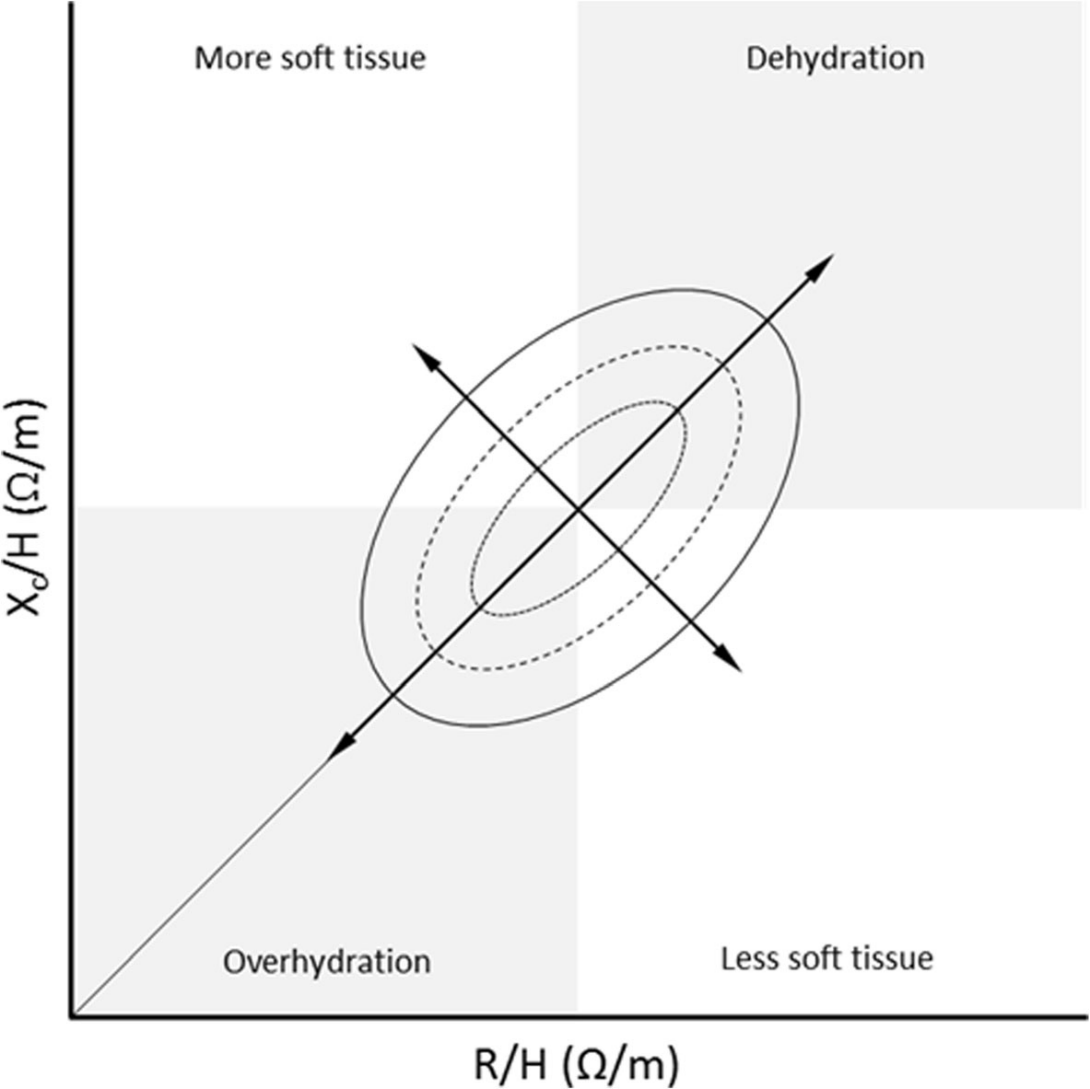
Interpretation of the BIVA nomogram. Reference values are plotted as so-called tolerance ellipses in the coordinate system with the 50th (∙∙∙∙), 75th (−---) and 95th (──) vector percentile of the healthy reference population. Values outside of the 95th percentile are considered abnormal. The position and length of the vector provides information about disease status and cell membrane function. The length of the vector is related to high or low R values, i.e. dehydration (quadrant 1) and overhydration (quadrant 3), respectively. A migration sideways of the vector due to high or low X_C_ indicates increase (quadrant 2) or decrease (quadrant 4) of dielectric mass (membranes and tissue interfaces) of soft tissues. The same interpretation applies when BIVA plots are prepared based on Z scores. The division into quadrants is meant as an indication only, not as sharp distinctions

The BIVA approach [17, 25] plots the loci of R and X_C,_ standardized by the height (H), i.e., R/H and X_C_/H (in Ω/m), as a bivariate vector in a nomogram. Data for specific populations, notably normative control data, are summarized as tolerance ellipses (see Fig. 1) against which patient data may be compared. Where replicate measurements are available as in the present study, for the NS patients before and after treatment, the different vectors (loci) can be shown in the same plot to visualize any change, such as change in position relative to the tolerance ellipses.

To better focus on deviation from normative reference values, Z scores were calculated. For a normal material with a mean value (μ) and a standard deviation (σ), the Z score of an observed value X is:

2 $$ \mathrm{Z}\ \mathrm{score}=\frac{\mathrm{X}-\upmu}{\upsigma} $$

In the present paper, Z scores are denoted Z_S_ (no unit) to avoid confusion with the accepted symbol for impedance (Z, in Ω). By definition Z_S_(μ) = 0, while a deviation by, for example 2 standard deviations from the mean value, corresponds to Z_S_ = 2.

The BIVA plots were transformed from raw plots to Z_S_ plots by plotting the Z_S_ values instead of the R and X_C_ values. In a BIVA plot transformed to Z_S_, the tolerance ellipses have center at (0,0), but will still be non-circular because R and X_C_ are not independent variables.

In this study, BIVA plots were prepared from R and X_C_ (raw impedance data), measured at either 50 kHz or *f*_c._ These BIVA plots will be referred to as BIVA_RXc_, with the corresponding parameters: R_50_/H and X_C50_/H, or R_*f*c_/H and X_C*f*c_/H. Correspondingly, BIVA plots transformed to Z scores will be referred to as BIVA_Zs_, with the corresponding parameters: Z_S_(R_50_/H) and Z_S_(X_C50_/H), or Z_S_(R_*f*c_/H) and Z_S_(X_C*f*c_/H).

#### Impedance ratio (IR)

The theory behind the use of IR’s is that at a sufficiently high frequency electrical current can penetrate the cell membrane, and provide information about the TBW, whereas at a low frequency the membrane is essentially impermeable to the electrical current, and only ECW can be measured [26]. While the ideal high and low frequencies are infinite and zero respectively, finite frequencies must be used in practice. The frequency pair 200 kHz and 5 kHz is commonly used [5, 12]. Impedance will be lower at 200 kHz than at 5 kHz, and IR_200/5_ will thus be a number below 1.00. An IR_200/5_ approaching 1.00 is deemed to indicate fluid overload and poor cellular health [12]. A high ratio is thus an expression that the resistance to electrical current in the body is reduced as a consequence of expansion of the ECW and/or a detriment of the normal function of the cell membrane.

The IR (no unit) was calculated from the impedance Z (in Ω) as follows:

3 $$ {\mathrm{IR}}_{\mathrm{high}/\mathrm{low}}=\frac{\mathrm{Z}\ \mathrm{at}\ \mathrm{high}\ \mathrm{kHz}}{\mathrm{Z}\ \mathrm{at}\ \mathrm{low}\ \mathrm{kHz}.} $$

The ratios considered were IR_200/5_ and IR_*f*c/5_, i.e., both the common choice and investigation of *f*_c_ as the high frequency.

### BIA measurements

BIA measurements were undertaken as far as possible following previously reported standardized testing and reporting procedures [7, 27]. Briefly, the protocol was as follows.

Participants were not fasting with no restrictions on voiding but had been requested to refrain from intense physical exercise four hours prior to study.

Measurements were performed in an electrically neutral environment with participants lying supine on a non-conductive surface (hospital bed/examination table). Participants were rested in the supine position for 5 min before and during measurement. Participants remained clothed with only hands and feet uncovered with the body positioned with the arms and legs abducted at a 30–45 degree angle from the trunk. Skin surface ECG-style gel electrodes with an area > 4 cm^2^ (single-Tab. 292-STE, ImpediMed, Brisbane QLD, Australia) were used: voltage (sensor) electrodes were applied at midline (electrode centre-to-centre) between the prominent bone prominences on the dorsal surface of the wrist (ulna and radius), and ankle (medial and lateral malleoli). The current (source) electrodes were placed with the midline 5 cm distal to these positions using a purpose-designed spacer. Where the hands or feet were too small to obtain this separation, the current electrodes were placed as distally as possible on the hand (but not on the fingers or toes), and the voltage electrodes were placed with the midline 5 cm proximal to this position [28]. To secure proper electrode-to-skin adherence and to minimize skin contact impedance, the skin was cleaned with alcohol (ethanol 75%) prior to the placement of electrodes.

Whole body (wrist to ankle) impedance was measured using a Xitron 4200, HYDRA BIS device, tested with an electronic verification module (TS4201) weekly according to manufacturer’s instruction, (Xitron Technologies, San Diego, CA, USA). This device measures the electrical parameters R and X_C_ at 50 discrete frequencies in the range from 5 to 1000 kHz.

It was ensured that the device cables were not touching the subject, the subjects’ parents, the ground, metal objects, routed near high voltage equipment, strong electrical or magnetic fields, and that the cables were not intertwined. All measurements were made by the same trained operator. Furthermore, the measurements were made in triplicate with electrodes remaining in place between measurements, made at room temperature (21^o^ to 25^o^ C) and performed between 08:30 and 15:30. The total measurement time was 7 min, covering patient preparation and impedance measurements.

Data were analysed and screened for data quality using the ImpediMed SFB7 Multi-Frequency Analysis software (Bioimp Version 5.4.0.3, Brisbane QLD, Australia) as described previously [29]. Precision of measurement was assessed by the percentage coefficient of variation (CV% = SD/mean ∙ 100%) and deemed acceptable for all groups: CV(R_50_ and R_*f*c_) ≤ 0.5% and CV(X_C50_ and X_C*f*c_) ≤ 3.1%.

### Statistical data analysis

Results are presented as mean ± standard deviation (SD), after test for normality, using Q-Q plots and the Shapiro-Wilk test.

To determine differences in impedance data between patients with active NS and the same patients at remission (ANS* group vs. NSR group), a paired two-tailed Student’s t-test was used. To compare impedance data between patients with active NS and at remission with controls (ANS group vs. HC group and NSR group vs. HC group), an unpaired two-tailed t-test was used.

The statistical software MedCalc® (MedCalc Software, Ostend, Belgium) was used to prepare all statistical tests and graphical illustrations.

## Results

### Participant characteristics

Comparison between all the participants enrolled in the study (Additional file 1: Table S1) showed no significant differences in any parameters between the NS and control participants and between the NS sub-groups.

All ANS patients (Additional file 1: Table S2) had normal or near normal renal function and four of eight patients were hypertensive at admission.

Repeat impedance measurements at remission were only possible in five of the patients: two of the ANS patients did not recover due to repeated relapses, and one was transferred to another hospital.

### Bioimpedance parameters

Impedance data and statistics are presented in Table 2. The main findings are summarized in the following.

**Table 2:** Measured impedance data and statistics for the included groups

Parameter	ANS	ANS*	NSR	HC	ANS* vs. NSR (paired)	ANS vs. HC (unpaired)	NSR vs. HC (unpaired)
*Raw impedance data*							
*f*_c_ (Hz)	144.5 ± 43.1	155.8 ± 44.5	96.8 ± 43.9	93.2 ± 29.2	*p* < 0.01	*p* < 0.05	*p* > 0.05
R_50_ (Ω)	421.5 ± 44.7	399.3 ± 41.0	663.1 ± 61.5	721.9 ± 65.3	*p* < 0.001	*p* < 0.001	*p* > 0.05
R_*f*c_ (Ω)	403.4 ± 40.9	382.1 ± 37.4	639.8 ± 58.2	694.7 ± 62.7	*p* < 0.001	*p* < 0.001	*p* > 0.05
X_C50_ (Ω)	22.1 ± 5.4	18.6 ± 3.4	63.0 ± 11.9	66.9 ± 8.1	*p* < 0.01	*p* < 0.001	*p* > 0.05
X_C*f*c_ (Ω)	24.2 ± 5.7	21.2 ± 5.2	72.4 ± 11.2	73.8 ± 10.1	*p* < 0.001	*p* < 0.001	*p* > 0.05
Z_5_ (Ω)	442.1 ± 47.9	416.2 ± 42.8	733.9 ± 65.1	794.9 ± 71.6	*p* < 0.001	*p* < 0.001	*p* > 0.05
Z_200_ (Ω)	396.8 ± 41.2	378.0 ± 39.4	603.7 ± 59.6	656.8 ± 59.9	*p* < 0.001	*p* < 0.001	*p* > 0.05
Z_*f*c_ (Ω)	404.3 ± 41.1	382.8 ± 37.5	643.1 ± 58.4	698.6 ± 63.0	*p* < 0.001	*p* < 0.001	*p* > 0.05
*PA (degrees)*							
PA_50_	3.0 ± 0.6	2.7 ± 0.4	5.5 ± 1.1	5.3 ± 0.5	*p* < 0.01	*p* < 0.001	*p* > 0.05
PA_*f*c_	3.4 ± 0.6	3.1 ± 0.5	6.5 ± 1.0	6.1 ± 0.6	*p* < 0.01	*p* < 0.001	*p* > 0.05
*BIVA*_*RXc*_ *(Ω/m)*							
R_50_/H	356.9 ± 87.6	337.2 ± 91.8	555.8 ± 166.9	579.4 ± 94.9	*p* < 0.01	*p* < 0.001	*p* > 0.05
R_*f*c_/H	341.1 ± 81.0	322.4 ± 85.6	530.6 ± 142.3	556.5 ± 83.4	*p* < 0.01	*p* < 0.001	*p* > 0.05
X_C50_/H	18.4 ± 4.9	15.4 ± 3.4	50.6 ± 7.6	58.8 ± 9.5	*p* < 0.001	*p* < 0.001	*p* > 0.05
X_C*f*c_/H	20.5 ± 6.6	17.9 ± 6.9	58.7 ± 10.8	58.7 ± 10.8	*p* < 0.001	*p* < 0.001	*p* > 0.05
*BIVA*_*Zs*_ *(no unit)*							
Z_S_(R_50_/H)	−2.3 ± 0.9	−2.6 ± 1.0	−0.3 ± 1.7	0.0 ± 1.0	*p* < 0.01	*p* < 0.001	*p* > 0.05
Z_S_(R_*f*c_/H)	−2.6 ± 1.0	−2.8 ± 1.0	−0.3 ± 1.7	0.0 ± 1.0	*p* < 0.01	*p* < 0.001	*p* > 0.05
Z_S_(X_C50_/H)	− 4.5 ± 0.6	−4.9 ± 0.4	−0.4 ± 1.0	0.0 ± 1.0	*p* < 0.001	*p* < 0.001	*p* > 0.05
Z_S_(X_C*f*c_/H)	−4.0 ± 0.7	−4.3 ± 0.7	0.0 ± 1.1	0.0 ± 1.0	*p* < 0.001	*p* < 0.001	*p* > 0.05
*IR (no unit)*							
IR_200/5_	0.90 ± 0.02	0.91 ± 0.01	0.82 ± 0.03	0.83 ± 0.02	*p* < 0.01	*p* < 0.0001	*p* > 0.05
IR_*f*c/5_	0.92 ± 0.01	0.92 ± 0.01	0.88 ± 0.01	0.88 ± 0.01	*p* < 0.001	*p* < 0.001	*p* > 0.05

Data are means ± SD. For group and impedance descriptions, see Table 1. H: height, Z: impedance (Z^2^ = R^2^ + X_C_^2^), Z_S_ = Z score

For group and impedance descriptions, see Table 1; Z_S_ = Z score; A Student’s t-test was used to determine differences between the groups with statistical significance set at a *p*-value < 0.05

#### Phase Angle

The absolute and relative (in brackets) mean differences of the PA values for the ANS patients compared to the HC were − 2.3 degrees (− 43.4%) for PA_50_ and − 2.7 degrees (− 44.3%) for PA_*f*c_. For the HC group, PA_50_ values showed to be comparable with earlier reported reference values (range: 5–7 degrees) for healthy children, adolescents and adults [13, 30], confirming their use as control group in this study.

For both PA_50_ and PA_*f*c_, significant differences were found between the NS children and the same children at remission (ANS* vs. NSR, both *p*-values < 0.01) as well as between the NS children and healthy children (ANS vs. HC, both *p*-values < 0.001). Children in remission had similar results to the healthy children (NSR vs. HC, both *p*-values > 0.05). The relationship between PA_50_ in relation to age and weight showed (Additional file 1: Fig. S2) that values for the ANS patients were located outside the confidence intervals of the HC at baseline, but for those participants for whom data at remission was available (i.e. the NSR group), data were now located within or very close to the range of control data.

#### BIVA

The absolute and relative (in brackets) mean differences of the BIVA_RXc_ parameters for the ANS patients compared to the HC were − 222.5 Ω/m (− 38.4%) for R_50_/H, − 35.0 Ω/m (− 65.5%) for X_C50_/H, − 215.5 Ω/m (− 38.4%) for R_*f*c_/H and − 38.3 Ω/m (− 65.1%) for X_C*f*c_/H. For the HC group,

The absolute mean differences of the BIVA_Zs_ parameters for the ANS patients compared to the HC were − 2.3 for Z_S_(R_50_/H), − 4.5 for Z_S_(X_C50_/H), − 2.6 for Z_S_(R_*f*c_/H) and − 4.0 for Z_S_(X_C*f*c_/H).

For both BIVA_RXc_ and BIVA_Zs_, significant differences were found between the NS children and the same children at remission (ANS* vs. NSR, all *p*-values < 0.01) as well as between the NS children and healthy children (ANS vs. HC, all *p*-values < 0.001). Children in remission had similar results to the healthy children (NSR vs. HC, all *p*-values > 0.05).

**Fig. 2:**
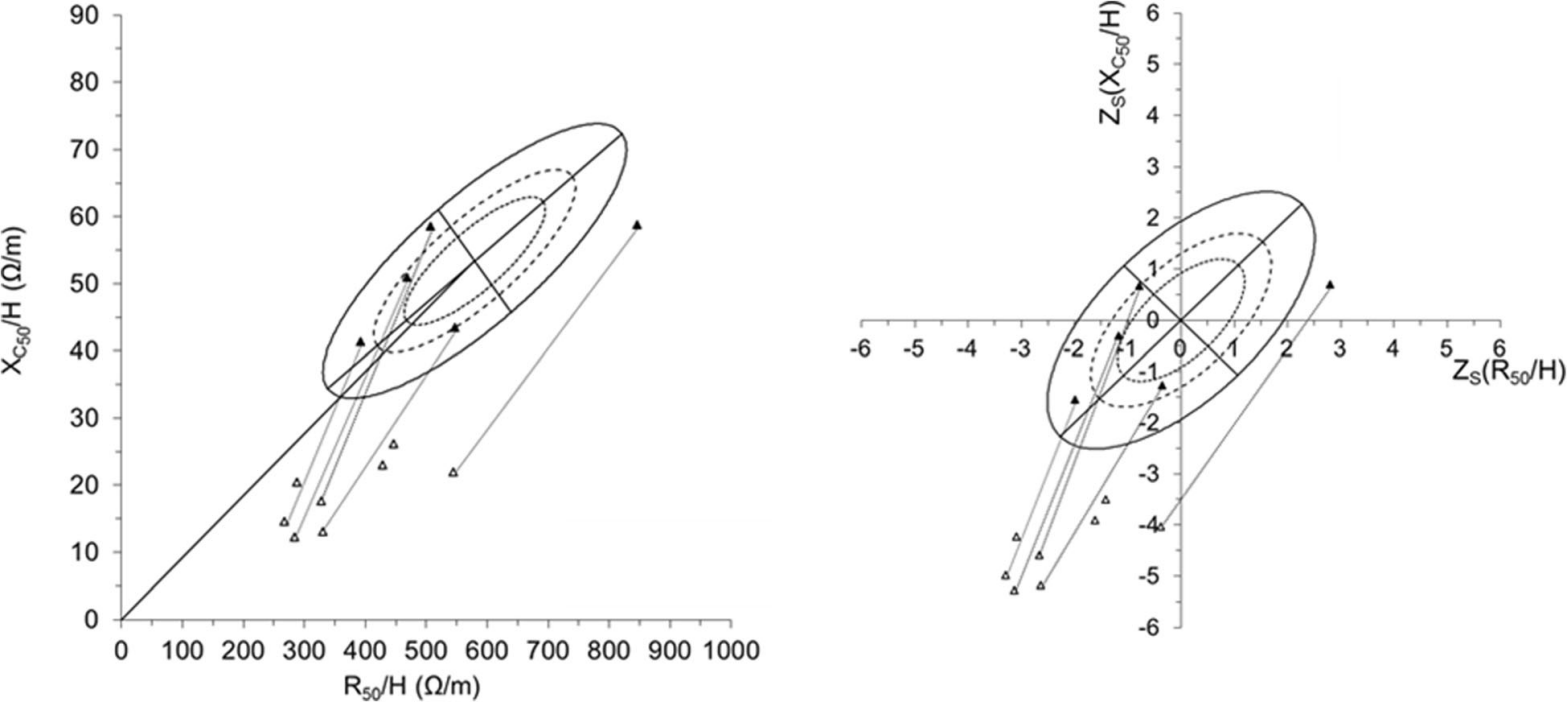
BIVA plots. BIVA_RXc_ and BIVA_Zs_ graphs calculated on the basis of 50 kHz. Reference ranges of the HC are indicated as 50th (∙∙∙∙), 75th (−---) and 95th (──) tolerance ellipses. ∆: ANS, ▲: NSR. To identify impedance changes, the plots are connected with dotted lines for the five ANS patients measured in the period of remission. For group and impedance descriptions, see Table 1

Figure 2 shows the BIVA graphs measured at 50 kHz for all the groups (ANS/ANS* and NSR as points, HC as reference ellipses). Graphs measured at the characteristic frequency *f*_c_ were very similar and are not shown. From the graphs it appears that the ANS patients were found outside the reference ellipse and in the quadrant that represents overhydration. At remission, vectors had moved along a trajectory that had returned the ANS participants to within the reference ellipse except for one ANS patient that was now located in the quadrant indicative of dehydration.

#### Impedance ratios

The absolute and relative (in brackets) mean differences for the ANS patients compared to the HC were 0.07 (8.4%) for IR_200/5_ and 0.04 (4.5%) for IR_*f*c/5_.

For both ratios studied, NS children were significantly different from the same children at remission (ANS* vs. NSR, both *p*-values < 0.01), and NS children were different from healthy children (ANS vs. HC, both *p*-values < 0.01), while no significant differences were found between children in remission and healthy children (NSR vs. HC, both *p*-values > 0.05).

**Fig. 3:**
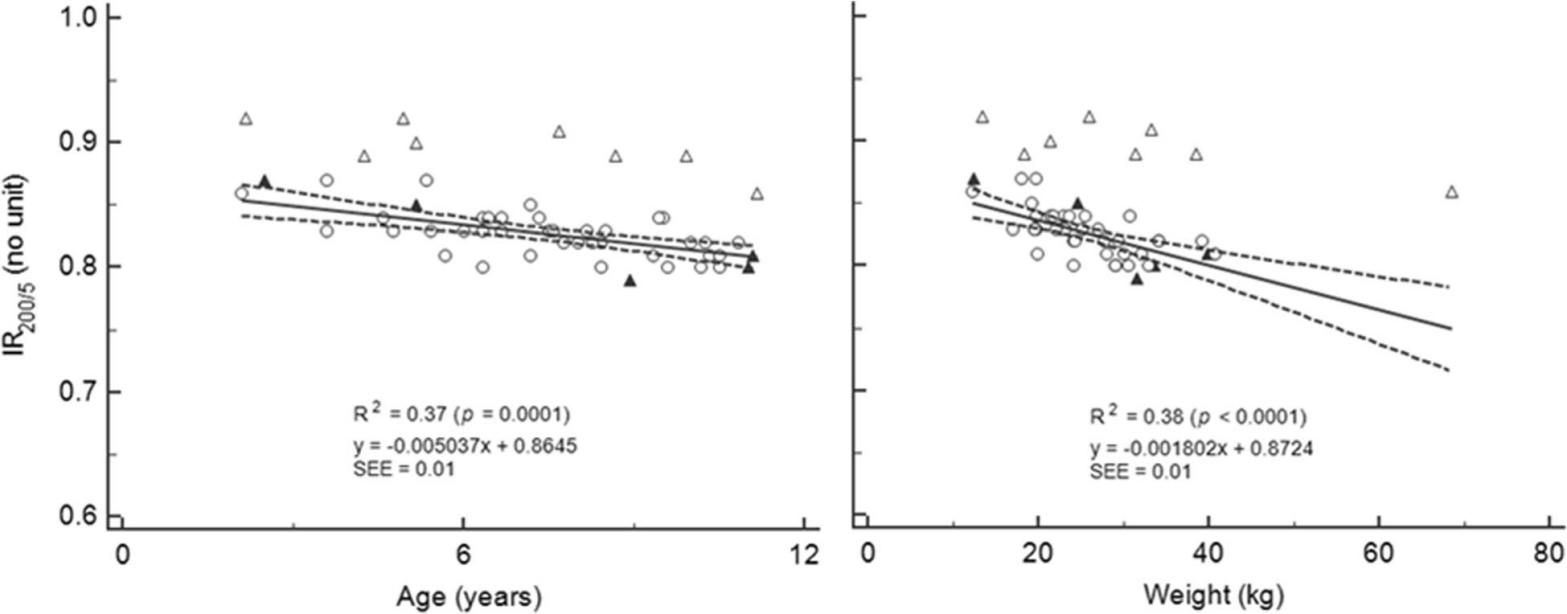
Relationship between impedance ratios and participants’ age and weight. ∆: ANS, ▲: NSR, ○: HC; For group and impedance descriptions, see Table 1; Regression lines are based on the HC data. Dashed lines are 95% confidence intervals for regression lines

The relationship between the IR_200/5_ and age and weight for all three groups are presented in Fig. 3. The ratios for the ANS patients were located outside the confidence intervals of the HC at baseline, but moved to be located within or very close to the normal range at remission. One ANS patient was notable for being an apparent outlier for weight (Fig. 3); this patient’s weight was at the 100th percentile from the ‘WHO child growth standards’ [31]).

## Discussion

This study has demonstrated that BIA is capable of distinguishing NS patients from healthy controls and is capable of tracking recovery of patients at remission. In contrast to earlier BIA studies in children with NS, where prediction equations have been used [32, 33], the current study, and a recently published bioimpedance study in the same group of children [34], used raw BIA data free of the assumptions that underlie prediction equations for body water volumes. These assumptions, for example, constant hydration, are questionable in many clinical situations, and provide results open to misinterpretation [12, 35].

Three approaches to the use of raw impedance data were considered: PA, impedance ratios and BIVA plots. It is appropriate to consider the relative advantages and disadvantages of each.

Low PA values have been previously associated with ill health. PA values below 3.0 degrees were found to be an independent predictor of poor survival in haemodialysis patients [36]. This value showed to be in agreement with the mean value of 3.0 degrees observed in the ANS patients. Similarly, low PA values (male: 4.21 degrees; female: 4.38 degrees) have been observed in children suffering from sickle cell disease (SCD) [37] and malnutrition (2.4 degrees) [38].

In contrast to PA, BIVA plots make it possible to differentiate between two identical PA values that may represent two different physiological conditions (cf. Figure 1) as well as the relation between X_C_ and R which is subsumed into the single PA value. A disadvantage of BIVA plots is, however, the time required to prepare such plots for pediatric patients. Although some commercially available BIA devices provide BIVA plots in their software, unfortunately they do not include reference ranges for pediatric populations.

Compared to PA and BIVA (both calculated at 50 kHz), impedance ratios provide information related to body water distribution, i.e. ECW relative to TBW. In previous studies, IR has proven to be a more sensitive indicator of malnutrition than PA [39]. To be clinically useful, interpretation of IRs requires normative data (cut-points) obtained in comparable populations; unfortunately, at present, no normative data are available for the pediatric population. In healthy adults, mean IR_200/5_ cut-points of between ≤0.78 and ≤ 0.82, respectively, have been reported as limits for normal IR_200/5_ [39], and reference cut-points based on ethnicity for healthy adults have been prepared by NHANES [30]. For comparison, the mean IR_200/5_ was 0.83 in our pediatric HC group, i.e. slightly above the adult cut-point.

Studies in adults have demonstrated that ratios progressively closer to 1.00 are linked to fluid overload, poor cellular health and poor clinical outcomes [12]; by comparison a mean ratio of 0.90 was observed in the ANS patients. The rationale for calculating IR at frequencies 5 and 200 kHz, is that 5 kHz is sufficiently low to provide an accurate measure of ECW while 200 kHz is sufficiently high that a large proportion of the electrical current penetrates the cell membrane and thus will provide a more accurate measure of TBW [12, 40].

The secondary aim of the present study was to explore whether there was advantage in using PA, BIVA and IR measurements obtained at *f*_c,_ as alternative to the widely used 50 kHz. Even though *f*_c_ has been suggested as the optimal frequency for impedance measurements in humans [5], our data, however, do not support this contention, since the patient and control groups were equally well separated by measurements at either frequency. This observation is consistent with the finding that measurements of impedance at *f*_c_ are no better predictors of body composition than at 50 kHz [40]. It should be emphasized that using a wrist to ankle measurement protocol, as used in this study, *f*_c_ is the average value of the individual *f*_c_ values for all tissues within the conductive route. All tissues display different and unique *f*_c_’s that depend upon their physical structure. Whole body impedance measurements are predominantly determined by the impedance of skeletal muscle tissue [41]. Thus it is possible that the increased *f*_c_ observed in the ANS patients is indicative of changes in skeletal muscle; a hypothesis consistent with NS being a systemic condition.

Limitations of the present study were the low number of patients enrolled, which is a consequence of the low incidence of the disease with only around 2 new cases per 100,000 [42]. Despite the risk that the small number of patients is not representative of NS patients in general, we do not consider this to be a serious problem as they exhibited the same clinical characteristics as previous patients in the clinic.

BIVA plots for the reference population are normally adjusted for age, BMI and gender. This was not possible in this study due to the limited number of controls enrolled.

An adjusted BIVA plot is likely to exhibit narrower normal ranges, i.e. smaller tolerance ellipses. Figure 2 demonstrates that the BIVA data of the ANS children were clearly outside the normal tolerance ellipses, tighter normal ranges would amplify these differences.

Generally, no attempt was made to adjust for sex differences, again due to small sample size, even though significant differences between the sexes regarding relative fat mass and lean body mass (LBM) even when adjusted by height have been found in studies in healthy children [43].

## Conclusion

This study demonstrated that PA, BIVA and IR may be clinically useful to monitor changes in disease status in pediatric NS patients. NS is characterized by leakage of large amounts of proteins from the kidneys into the urine, with consequent hypoalbuminemia and oedema formation. Sodium retention is the major clinical feature of NS and the primary cause of the oedema formation. PA and BIVA measurements can be obtained using simple inexpensive single frequency bioimpedance devices. Multi-frequency devices are required if IR is applied for additional information on the relative sizes of body water compartments, i.e. ECW and TBW. In order to increase the clinical utility of the approaches considered here, standardized population-specific reference data from childhood to puberty are required.

The present results are promising, in that they demonstrate the potential of BIA as an alternative clinical tool to repeated daily weighing as a measure of fluid overload. Weight change is a poor prognostic indicator since premorbid weight is often influenced by recall bias from the carers of the child and the weight of a child will change over time, especially in NS patients treated with prednisolone which enhances appetite markedly.

Finally, although the present data do not provide a physiological mechanism for changes in disease status in NS, changes in tissue reactance imply changes in cell membrane function with consequent impacts upon tissue water distribution. Further studies are required to confirm this suggestion.

## Additional file

Additional file 1: **Figure S1.** Raw impedance data in the impedance plane. **Figure S2.** Relationship between phase angle and participants’ age and weight. **Table S1.** Characteristics of the study subjects. **Table S2.** Clinical data for the ANS patients. (PDF 201 kb)

## Data Availability

The dataset used during the current study is available from the corresponding author on reasonable request at the following address: stebra@rm.dk

## Abbreviations

ANS: Active nephrotic syndrome (study group)
BIA: Bioimpedance analysis
BIS: Bioimpedance spectroscopy
BIVA: Bioimpedance vector analysis
ECW: Extra-cellular water
*f*_*c*_: Characteristic frequency
H: Person height in cm
HC: Healthy controls (control group)
ICW: Intra-cellular water
IR: Impedance ratio
NS: Nephrotic syndrome
NSR: Nephrotic syndrome in remission (study group)
PA: Phase angle
R: Electrical resistance
TBW: Total body water
X_C_: Capacitive part of the electrical impedance (Z)
Z: Electrical impedance
Z score, Z_S_: Number of standard deviations, cf. equation (2), not to be confused with electrical impedance Z
