# Supplementary material for: An evaluation of phase angle, bioelectrical impedance vector analysis and impedance ratio for the assessment of disease status in children with nephrotic syndrome
Source: BMC Nephrol. 2019 Aug 22;20:331. doi: 10.1186/s12882-019-1511-y (PMC6704501; doi:10.1186/s12882-019-1511-y)
Supplement: Supplementary file 1 — Figure S1. Raw impedance data in the impedance plane. Figure S2. Relationship between phase angle and participants’ age and weight. Table S1. Characteristics of the study subjects. Table S2. Clinical data for the ANS patients. (PDF 201 kb) [file 12882_2019_1511_MOESM1_ESM.pdf]

Figure S1: Raw impedance data in the impedance plane.

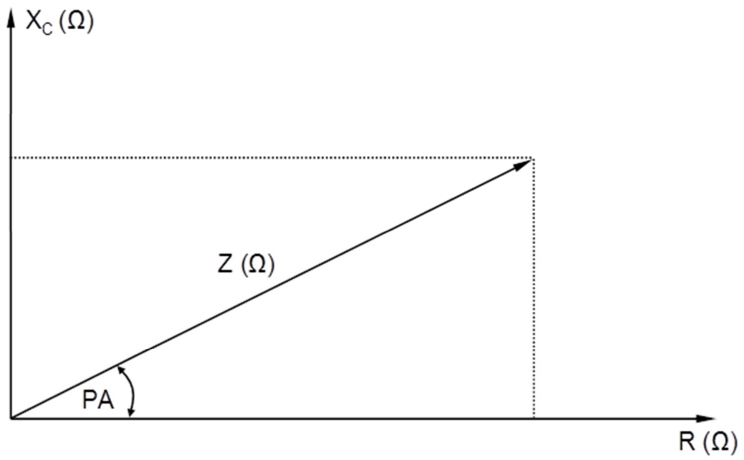

The relationship between capacitive reactance (X_C), resistance (R), impedance (Z) in Ohms (Ω) and phase angle (PA) in degrees.

Figure S2: Relationship between phase angle and participants' age and weight.

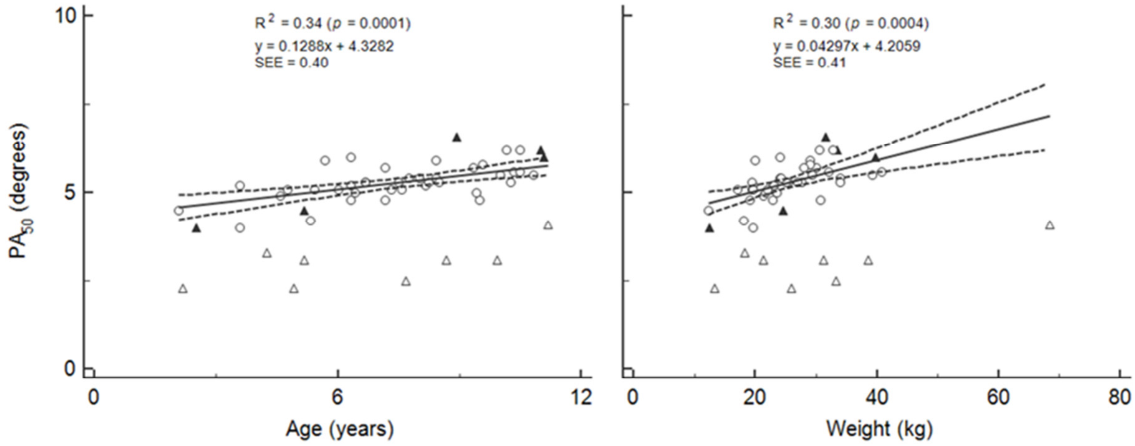

Δ: ANS, ▲: NSR, ○: HC; For group and impedance descriptions, see Table 1; Regression lines are based on the HC data.

Table S1: Characteristics of the study subjects.

Group /Parameter	Sex	Age (years)	Study weight (kg)	Height (cm)	BMI (kg/m^2)
ANS	7 M: 1 F	6.7 ± 3.1	31.3 ± 17.1	120.7 ± 21.1	20.1 ± 4.6
ANS*	4 M: 1 F	6.8 ± 3.1	28.5 ± 9.6	120.3 ± 21.8	19.1 ± 1.5
NSR	4 M: 1 F	7.7 ± 3.8	28.3 ± 10.4	126.1 ± 26.2	17.3 ± 2.0
HC	23 M: 15 F	7.5 ± 2.2	25.3 ± 6.2	126.4 ± 14.2	15.6 ± 1.2

Data are means ± SD; For group definitions (ANS, ANS*, NSR, HC), see Table 1; BMI: body mass index.

Table S2: Clinical data for the ANS patients.

**Patients (No.) /Parameters**	**Sex**	BP (mmHg)	HT[†] (yes/no)	Pulse (bpm)	P-Albumin (g/L)	P-sodium (mmol/L)	P-potassium (mmol/L)	P-creatinine (µmol/L)	eGFR[‡] (ml/min/1.73m^2)	B-Hgb (mmol/L)
Age: 1-4 years										
1*	M	110/70	yes	109	10	139	3.5	18	177	8.5
2	M	88/62	no	97	10	141	3.6	30	123	7.9
3*	M	109/68	no	92	9	137	5.5	24	167	8.1
Age: 5-11 years										
4	M	109/71	yes	155	7	131	3.5	21	195	8.5
5*	F	130/93	yes	77	9	132	4.9	71	65	9.3
6*	M	116/77	no	104	9	129	5.0	50	94	9.4
7*	M	133/73	yes	99	9	137	3.8	56	94	8.7
8	M	109/56	no	102	4	127	3.9	50	108	10.6

Patients in the subgroup ANS, which were re-studied at remission, see Table 1.
BP: blood pressure; HT: hypertension; Hgb: Hemoglobin.
[†]Hypertension defined as blood pressure above the 95% percentile for high and gender.
[‡]eGFR estimated by the Schwartz formula[1]. Reference ranges: P-Albumin (37-48), P-Sodium (137-145), P-potassium (3.5-4.6), B hemoglobin (6.5-8.9).

[1] Schwartz GJ, Muñoz A, Schneider MF, Mak RH, Kaskel F, Warady BA, m.fl. New Equations to Estimate GFR in Children with CKD. J Am Soc Nephrol. 3. januar 2009;20(3):629–37.1).
